# Commentary: Comparison of an Innovative Rehabilitation, Combining Reduced Conventional Rehabilitation With Balneotherapy, and a Conventional Rehabilitation After Anterior Cruciate Ligament Reconstruction in Athletes

**DOI:** 10.3389/fsurg.2021.665748

**Published:** 2021-06-23

**Authors:** Som P. Singh, Kiera G. Borthwick, Fahad M. Qureshi

**Affiliations:** ^1^School of Medicine, University of Missouri-Kansas City, Kansas City, MO, United States; ^2^Department of Neurosciences, Washington & Lee University, Lexington, VA, United States

**Keywords:** anterior cruciate ligament, balneotherapy, rehabilitation, sports injury, hydrotherapy

## Introduction

Ruptures of the anterior cruciate ligament (ACL) are relatively common in sporting activities, especially in sports that require frequent pivoting and rotation of the lower extremities. Previous literature has well-established a direct correlation between terms of the frequency of pivoting movements and the likelihood of ACL injury (1). Sports with the highest occurrences of pivoting movements require frequent changes of direction which include soccer, basketball, and handball. Contrastingly, sports that only require straight forward movements have very limited pivoting movements and changes of direction which include running and weight lifting (1–3).

Moreover, ACL repair is a major focus of orthopedics. The ACL belongs to a class of ligaments with a poor blood supply, thereby making spontaneous angiogenesis-based repair more difficult and prolonging natural healing without surgical reconstruction (3). ACL reconstruction is a relatively successful treatment and results in an increased activity and a reduced need for subsequent surgeries. However, only 65% of individuals return to the same competitive level of sport after ACL reconstruction surgery (2–4). Therefore, it is evident that there is a need to continue improving patient outcomes, specifically through rehabilitations (4, 5). Of note, hydrotherapy is the methodology of interest in this commentary. This methodology uses the physical properties of water, such as gravitation compensation in rehabilitation protocols (6), and is a potential area of focus for improved patient outcomes following ACL reconstruction.

## Improved Functional Outcomes With Hybrid Land and Water Therapy

In a recent study by Peultier-Celli et al. (6), researchers implemented a unique combination of traditional and aquatic balneotherapy rehabilitation programs in amateur and professional athletes after ACL reconstruction surgery. All participants in the study followed the conventional rehabilitation for the first 2 weeks. At 2 weeks post-operative, participants were randomly assigned to either continue the traditional rehabilitation or begin a new protocol involving aquatic rehabilitation. After 3 weeks of the newly assigned protocols, all participants began traditional rehabilitation again. Patients who underwent the hybrid rehabilitation protocol had an increased contribution of proprioception to balance and posture, relied less strongly on the contralateral leg for posture, had a higher quadriceps and hamstring strength, and could walk further distances under time constraints (5). Additionally, Peultier-Celli et al. noted multiple occurrences of pain reduction among the athletic cohort undergoing the experimental protocol.

## Discussion

Peultier-Celli et al. integrated a combination of both land and water therapies in their methodology. Prominent literature on hydrotherapy by Tovin et al. has demonstrated contradictory findings, in which hydrotherapy was not as effective as land therapy for building muscle strength (7). In fact, Tovin et al. stated, “rehabilitation in water is equally effective as on land for restoring knee [range of motion] and quadriceps femoris muscle strength, but not as effective in restoring hamstring muscle strength. Clinicians who wish to allow maximal weight bearing may find the adjunct of aquatic exercises useful.” It seems that traditional land protocols may be more effective for certain aspects of rehabilitation, like muscular strength. Water immersion alters the order of motor unit recruitment (8), which may impact the transferability of strengthening exercises to land environments. Despite this, the hybrid protocol creates the argument that hydrotherapy can limit limb overcompensation during rehabilitation. If limb overcompensation is limited, it is beneficial for athletes in lowering re-injury risks, which makes this Peultier-Celli et al. methodology worth investigating. With regard to the pre-operative and operative aspects, this study discussed the utilization of auto-graft tendons for ACL repairs as optimal, but it is also possible to have a more heterogeneous result due to the variety of fixations methods such as bone-patellar tendon-bone (BPTB) with interference screw in those pursuing a hybrid treatment regimen (5, 7).

Of note, the experimental protocol in this study focused on a cohort of athletes. While the hybrid methodology shows promise in these cohorts, a future analysis ought to be performed on non-athlete cohorts. For example, there is literature which shows improved metabolic effects after a balneotherapy regimen in those who have a greater body mass index (9), but do the effects of implementing a transition from a traditional to aquatic rehabilitation medium generate a different change among this cohort? If there is a greater improvement in metabolism through this hybrid style, it could potentially be considered to be included in the ACL rehabilitation guidelines among the morbid obese. Likewise, another avenue of investigation would be a further stratified cohort of athletes, potentially by sports (10, 11). Given the aforementioned difference in pivot movements, sports that have a higher occurrence of pivot movements could have a different panel of rehabilitation requirements, such as a potentially longer timeline for hybrid treatment compared to sports with a lower occurrence. Furthermore, Peultier-Celli et al.'s cohort shows promise for those undergoing isolated ACL reconstruction, and it would be beneficial to develop a similar study focusing on athletes who undergo a multiple ligament knee reconstruction as this area of surgery has a lack of evidence on rehabilitation compared to isolated ACL reconstructions (12, 13).

## Conclusion

In aggregate, the study by Peultier-Celli et al. establishes an innovative protocol for rehabilitation following ACL reconstruction. While there are various avenues for future investigation, the optimal direction for study ought to be on evaluating the Peultier-Celli et al.'s hybrid protocol on different athletic and non-athletic cohorts. If this area of literature continues to develop, this could be an encouraging inclusion to the current ACL reconstruction rehabilitation guidelines (14, 15).

## Author Contributions

All authors listed have made a substantial, direct and intellectual contribution to the work, and approved it for publication.

## Conflict of Interest

The authors declare that the research was conducted in the absence of any commercial or financial relationships that could be construed as a potential conflict of interest.
